# ASO Author Reflections: Indocyanine Green Fluorescence Sentinel Lymph Node Biopsy in Breast Cancer—An Alternative to Blue Dye?

**DOI:** 10.1245/s10434-023-13951-0

**Published:** 2023-07-20

**Authors:** Chu Luan Nguyen, Michael Zhou, Neshanth Easwaralingam, Jue Li Seah, Farhad Azimi, Cindy Mak, Carlo Pulitano, Sanjay Kumar Warrier

**Affiliations:** 1 Department of Breast Surgery, Chris O’Brien Lifehouse, Camperdown, NSW Australia; 2 Department of Surgery, Royal Prince Alfred Hospital, Camperdown, NSW Australia; 3 Department of Surgery, The University of Sydney, Camperdown, NSW Australia

## Past

Sentinel lymph node (SLN) biopsy with blue dye (BD) and/or radioisotope (RI) has become standard of care in clinically node-negative early breast cancer since the tracers were introduced in the early 1990s. Fluorescence-guided SLN biopsy using indocyanine green (ICG), a green fluorophore dye, was introduced in 2005 for breast cancer in an attempt to overcome drawbacks of BD and RI.^1^ Following injection of ICG into the breast, a near-infrared camera allows for visualization of subcutaneous lymphatic flow and lymph nodes in real time for axillary dissection.^2^

There is no consensus on how ICG will complement or potentially replace traditional methods of SLN mapping. This is due to the technique’s infancy and quality of current available data mostly consisting of comparative cohort studies.^3^ There is a lack of data on a direct comparison between gold standard dual technique of BD-RI versus ICG combined with RI (ICG-RI).

## Present

Our study compared 150 prospective patients with early breast cancer undergoing SLN biopsy using ICG-RI with 150 prior patients using BD-RI.^4^ The number of SLNs identified, rate of failed mapping, identification of metastatic SLNs, and adverse reactions between the two techniques were found to be equivalent. Cost-minimization analysis showed that ICG-RI cost an additional AU $197 per case in addition to the imaging system cost, which could be a significant limiting factor in the adoption of ICG technology.

## Future

We found that novel tracer combination, ICG-RI, provided an effective and safe alternative to gold standard dual tracer. The proviso was the higher expense associated with ICG. Increased uptake of fluorescence technology in the future could potentially lower costs of the imaging system. The combination of ICG-RI could represent a transition phase with ICG used as a sole tracer in future. Future multicenter randomized controlled trials evaluating ICG as single or dual tracer are needed to provide definitive information for change in practice.
